# High fitness levels attenuate the increased risk of heart failure due to low socioeconomic status: A cohort study

**DOI:** 10.1111/eci.13744

**Published:** 2022-01-14

**Authors:** Setor K. Kunutsor, Sae Young Jae, Timo H. Mäkikallio, Jari A. Laukkanen

**Affiliations:** ^1^ National Institute for Health Research Bristol Biomedical Research Centre University Hospitals Bristol NHS Foundation Trust and University of Bristol Bristol UK; ^2^ Musculoskeletal Research Unit Translational Health Sciences Bristol Medical School University of Bristol Learning & Research Building (Level 1) Southmead Hospital Bristol UK; ^3^ Diabetes Research Centre University of Leicester Leicester General Hospital Leicester UK; ^4^ Department of Medicine Central Finland Health Care District Hospital District Jyväskylä Finland; ^5^ 35010 Department of Sport Science University of Seoul Seoul Korea; ^6^ 3835 Department of Medicine University of Helsinki Helsinki Finland; ^7^ Department of Medicine South‐Karelia Central Hospital Lappeenranta Finland; ^8^ Institute of Public Health and Clinical Nutrition University of Eastern Finland Kuopio Finland; ^9^ Institute of Clinical Medicine Department of Medicine University of Eastern Finland Kuopio Finland

**Keywords:** cardiorespiratory fitness, cohort study, heart failure, risk factor, socioeconomic status

## 1 INTRODUCTION

Heart failure (HF) is a cardiovascular disease (CVD) outcome that is associated with high morbidity and mortality as well as high healthcare costs.^1^ Given that HF is the end stage of most CVDs, both conditions share common risk factors such as type 2 diabetes (T2D), hypertension, smoking and obesity.^2^ Socioeconomic status (SES) has been recognized to have a measurable and significant effect on cardiovascular health. It has been reported that low SES may confer a cardiovascular risk that is equivalent to conventional risk factors.^3^ Low SES has been shown to be a powerful and independent predictor of HF development and adverse outcomes.^4^ Biological, behavioural and psychosocial risk factors prevalent in socioeconomically deprived individuals are known to accentuate the relationship between low SES and cardiovascular outcomes such as HF.^3^ These include lower levels of education, unhealthy lifestyles such as excessive alcohol consumption, limited access to health care and higher prevalence of comorbid conditions.

The beneficial effects of regular physical activity (PA) and exercise in preventing vascular disease and promoting overall health are well established and documented. These benefits also extend to HF prevention.^5^ Though cardiorespiratory fitness (CRF) reflects habitual aerobic PA, it is a separate measure that captures the capacity of the cardiovascular and respiratory systems to supply oxygen to skeletal muscles during progressive PA or incremental exercise to volitional fatigue.^6^ The gold standard for CRF assessment is direct measurement of the highest attained oxygen consumption (VO2) during cardiopulmonary exercise testing. Similar to PA, high levels of CRF are strongly and independently associated with lower risk of vascular outcomes including HF.^7^, ^8^ The inverse associations between CRF and vascular outcomes have been reported to be stronger than that of traditional risk factors such as T2D and smoking; this has led to CRF being proposed as a vital sign.^9^ There is increasing evidence showing that higher levels of CRF can attenuate the adverse impact of other risk factors; for instance, we and others have previously shown that high CRF levels can attenuate the impact of risk factors associated with mortality,^10^ pneumonia^11^ and COVID‐19 hospitalization.^12^ Given the evidence, we hypothesized that high CRF levels would attenuate the increased risk of HF due to low SES. To explore this, we aimed to evaluate the joint effects of SES and CRF on the risk of incident HF using a population‐based prospective cohort of 1831 middle‐aged Finnish men without a history of HF at baseline. We also evaluated the separate associations of SES and CRF with the risk of HF to confirm previous evidence of these associations.

## 2 METHODS

Reporting of the study conforms to broad EQUATOR guidelines^13^ and was conducted according to STROBE (STrengthening the Reporting of OBservational studies in Epidemiology) guidelines for reporting observational studies in epidemiology (Appendix S1). The current analysis is based on the Kuopio Ischaemic Heart Disease (KIHD) risk factor study, a general population‐based prospective cohort study comprising of a representative sample of men aged 42–61 years recruited in eastern Finland. A detailed description of the study design, recruitment methods, risk marker assessment and physical examinations have been described previously.^8^ Baseline measurements were performed between 01 March 1984 and 31 December 1989. The research protocol was approved by the Research Ethics Committee of the University of Eastern Finland and written informed consent was obtained from all the participants. A self‐reported questionnaire was used to assess SES, which involved a summary index that combined factors such as income, education, occupational prestige, material standard of living and housing conditions. The composite SES index ranged from 0 to 25, with higher values indicating lower SES. Maximal oxygen uptake (VO_2max_) was used as a measure of CRF, which was assessed using a respiratory gas exchange analyser (Medical Graphics, MCG, St. Paul, Minnesota) during cycle ergometer exercise testing.^14^ We excluded men with a prevalent history of HF for the current analysis. We included all HF events that occurred from study entry through to 2018. The diagnostic classification of HF cases was coded according to the ICD‐10 codes.

Hazard ratios (HRs) with 95% confidence intervals (CIs) for HF were calculated using Cox proportional hazard models and these were adjusted for in three models: (Model 1) age; (Model 2) Model 1 plus systolic blood pressure (SBP), body mass index (BMI), heart rate, smoking status, history of T2D, history of coronary heart disease (CHD), total cholesterol, high‐density lipoprotein cholesterol (HDL‐C) and PA; and (Model 3) Model 2 plus mutual adjustment for each exposure. For consistency with previous reports,^10^, ^15^ the exposures (SES and CRF) were categorized into low and high levels based on their median cutoffs. The exposures were also modelled as continuous variables given evidence of linear relationships with HF risk using multivariable restricted cubic spline curves. Evaluation of the joint association of SES and CRF with HF risk was based on the following four combinations: high SES‐low CRF; low SES‐ low CRF; high SES‐high CRF and low SES‐high CRF. Tests of interaction were used to formally assess if the risk of HF due to one exposure is modified by the other exposure and vice versa. To put our findings into clinical context, we also calculated the number needed to treat (NNT) associated with high SES‐high CRF using the formula proposed by Altman and Anderson^16^: NNT (*t*) =1/[S_B_(*t*))^HR^ – S_B_(*t*)], where S_B_(*t*) denotes the Kaplan–Meir survival probability in the reference group (High SES‐Low CRF) at time *t* and HR refers to the Cox regression estimate comparing the exposure group with the reference group. Stata version MP 16 (Stata Corp, College Station) was employed for all analyses.

## 3 RESULTS

The overall mean (standard deviation, SD) age, SES and CRF of study participants at baseline was 52 (5) years, 8.26 (4.24) and 30.8 (7.9) ml/kg/min, respectively (Table 1). There were significant differences in baseline characteristics between low and high CRF groups.

**TABLE 1:** Baseline characteristics of study participants

Characteristics	Overall Mean (SD) or median (IQR) or *n* (%)	High CRF Mean (SD) or median (IQR) or *n* (%)	Low CRF Mean (SD) or median (IQR) or *n* (%)	*p*‐value
Socio‐economic status	8.26 (4.24)	7.54 (4.30)	8.98 (4.04)	<.001
Cardiorespiratory fitness (ml/kg/min)	30.8 (7.9)	36.9 (5.3)	24.6 (4.5)	<.001
Questionnaire/Prevalent conditions				
Age (years)	52 (5)	51 (5)	54 (4)	<.001
History of type 2 diabetes	58 (3.2)	13 (1.4)	45 (4.9)	<.01
Current smoking	591 (32.3)	248 (27.1)	343 (37.5)	<.01
History of CHD	379 (20.7)	95 (10.4)	284 (31.0)	<.01
Physical measurements				
BMI (kg/m^2^)	26.8 (3.5)	25.8 (2.8)	27.9 (3.7)	<.001
SBP (mmHg)	134 (16)	132 (15)	136 (18)	<.001
DBP (mmHg)	89 (10)	88 (10)	90 (11)	<.001
Heart rate (bpm)	62 (11)	61 (10)	64 (11)	<.001
Total PA (kj/day)	1171 (633–1963)	1260 (685–2023)	1071 (569–1888)	.011
Blood biomarkers				
Total cholesterol (mmol/l)	5.91 (1.08)	5.83 (1.08)	5.99 (1.07)	.001
HDL‐C (mmol/l)	1.29 (0.30)	1.34 (0.30)	1.23 (0.28)	<.001
Fasting plasma glucose (mmol/l)	5.31 (1.14)	5.13 (0.78)	5.48 (1.39)	<.001

Abbreviations: BMI, body mass index; CHD, coronary heart disease; CRF, cardiorespiratory fitness; DBP, diastolic blood pressure; HDL‐C, high‐density lipoprotein cholesterol; IQR, interquartile range; PA, physical activity; SBP, systolic blood pressure; SD, standard deviation.

**FIGURE 1:**
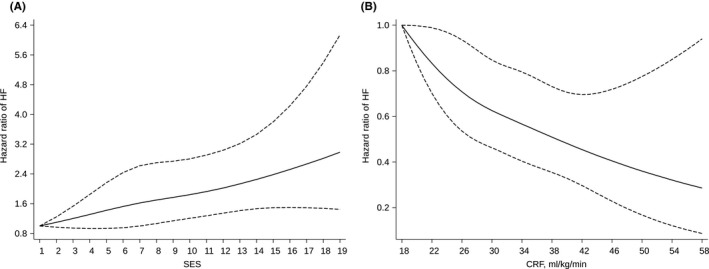
Restricted cubic splines of the hazard ratios of incident heart failure with socioeconomic status and cardiorespiratory fitness. (A) Socioeconomic status and HF risk; (B) Cardiorespiratory fitness and HF risk. CRF, cardiorespiratory fitness; HF, heart failure; SES, socioeconomic status. Models were adjusted for age, systolic blood pressure, body mass index, heart rate, smoking status, history of type 2 diabetes, history of coronary heart disease, total cholesterol, high‐density lipoprotein cholesterol, and physical activity

**FIGURE 2:**
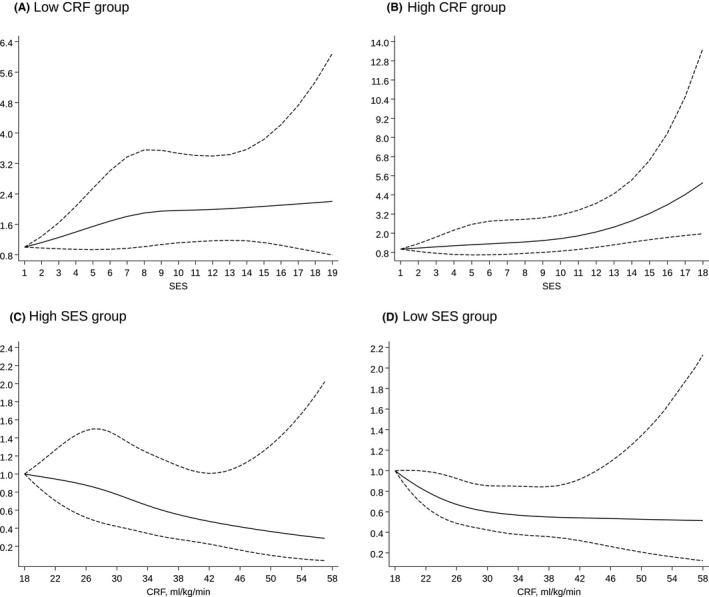
Restricted cubic splines of the hazard ratios of incident heart failure with socioeconomic status and cardiorespiratory fitness in subgroups of each exposure. (A) SES and HF risk in low CRF group (*p*‐value for nonlinearity =.43); (B) SES and HF risk in high CRF group (*p*‐value for nonlinearity =.48); (C) CRF and HF risk in high SES group (*p*‐value for nonlinearity =.86); (D) CRF and HF risk in low SES group (*p*‐value for nonlinearity =.43). CRF, cardiorespiratory fitness; HF, heart failure; SES, socioeconomic status. Models were adjusted for age, systolic blood pressure, body mass index, heart rate, smoking status, history of type 2 diabetes, history of coronary heart disease, total cholesterol, high‐density lipoprotein cholesterol and physical activity

During a median (interquartile range) follow‐up of 27.3 (18.6–31.2) years, 364 incident HF cases occurred. In an analysis adjusted for age, SBP, BMI, heart rate, smoking status, history of T2D, history of CHD, total cholesterol, HDL‐C and PA, low compared with high SES was associated with an increased risk of HF 1.43 (95% CI: 1.15–1.79), which remained similar on further adjustment for CRF. On adjustment for the confounders as above, high CRF was associated with a decreased risk of HF compared with low CRF 0.70 (95% CI: 0.55–0.89), which remained similar on additional adjustment for SES. There was evidence of significant associations when both exposures were modelled as continuous variables (Table 2). Restricted cubic spline curves with adjustment for age, SBP, BMI, heart rate, smoking status, history of T2D, history of CHD, total cholesterol, HDL‐C and PA showed that HF risk increased continuously with decreasing SES across the range 7–19 (*p*‐value for nonlinearity =.83) (Figure 1A), whereas HF risk decreased continuously with increasing CRF across the range 18–58 ml/kg/min (*p*‐value for nonlinearity =.79) (Figure 1B). The spline curves were qualitatively similar in subgroups of CRF and SES (Figure 2).

**TABLE 2:** Separate and joint associations of socioeconomic status and cardiorespiratory fitness with risk of heart failure

Exposure categories	Events/Total	Model 1		Model 2		Model 3	
		HR (95% CI)	*p*‐value	HR (95% CI)	*p*‐value	HR (95% CI)	*p*‐value
Socioeconomic status							
Per SD increase in SES	364/1831	1.41 (1.27–1.58)	<.001	1.27 (1.14–1.42)	<.001	1.27 (1.14–1.43)	<.001
High SES	146/922	ref		ref		ref	
Low SES	218/909	1.67 (1.35–2.07)	<.001	1.43 (1.15–1.79)	.001	1.41 (1.13–1.76)	.002
CRF (ml/kg/min)							
Per SD increase in CRF	364/1831	0.62 (0.55–0.70)	<.001	0.78 (0.68–0.90)	<.001	0.78 (0.68–0.89)	<.001
Low CRF	226/916	ref		ref		ref	
High CRF	138/915	0.49 (0.40–0.61)	<.001	0.70 (0.55–0.89)	.003	0.69 (0.55–0.88)	.002
Socioeconomic status and CRF (ml/kg/min) combination							
High SES‐Low CRF	88/390	ref		ref		NA	
Low SES‐ Low CRF	138/526	1.45 (1.11–1.89)	.007	1.32 (1.01–1.74)	.045	NA	
High SES‐High CRF	58/532	0.43 (0.31–0.60)	<.001	0.62 (0.43–0.89)	.009	NA	
Low SES‐High CRF	80/383	0.82 (0.61–1.11)	.21	1.01 (0.73–1.39)	.96	NA	

Note

Cut‐offs for SES and CRF were based on the median values.

Model 1: Adjusted for age.

Model 2: Model 1 plus systolic blood pressure, body mass index, heart rate, smoking status, history of type 2 diabetes, history of CHD, total cholesterol, high‐density lipoprotein cholesterol, and physical activity.

Model 3: Model 2 plus CRF for SES and SES for CRF.

Abbreviations: CHD, coronary heart disease; CI, confidence interval; CRF, cardiorespiratory fitness; HR, hazard ratio; NA, not applicable; ref, reference; SD, standard deviation; SES, socioeconomic status.

**FIGURE 3:**
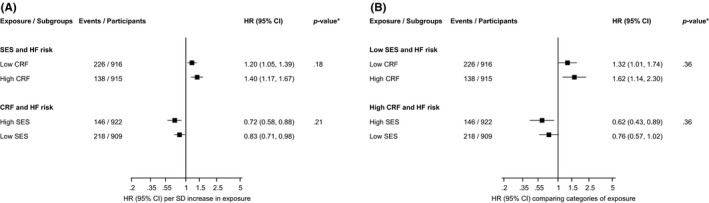
Interactions of the associations of socioeconomic status and cardiorespiratory fitness with incident heart failure. (A) Per 1 standard deviation increase in exposures (B) low/high vs. high/low categories of exposures. CI, confidence interval; CRF, cardiorespiratory fitness; HF, heart failure; HR, hazard ratio; SES, socioeconomic status; HRs are adjusted for age, systolic blood pressure, body mass index, heart rate, smoking status, history of type 2 diabetes, history of coronary heart disease, total cholesterol, high‐density lipoprotein cholesterol and physical activity; *, *p*‐value for interaction

In multivariable analysis, low SES‐low CRF was associated with an increased HF risk 1.32 (95% CI: 1.01–1.74), high SES‐high CRF with a decreased HF risk 0.62 (95% CI: 0.43–0.89), with no evidence of an association for low SES‐high CRF and HF risk 1.01 (95% CI: 0.73–1.39) when compared with men with high SES‐low CRF (Table 2). The association of SES with HF risk was not modified by CRF (*p*‐value for interactions >.10) and neither was the association between CRF and HF risk modified by SES (*p*‐value for interactions >.10), when both exposures were modelled as continuous or categorical variables (Figure 3).

The absolute risk reduction of HF associated with high SES‐high CRF was 0.21 during the entire duration of follow‐up, which translated into a NNT of 10 (95% CI: 6–35) to prevent one HF.

## 4 DISCUSSION

Our results based on a general population‐based prospective cohort study of middle‐aged to older Finnish men confirms the previously reported independent associations of low SES with increased HF risk and high CRF levels with lowered risk of HF. The associations were also potentially consistent with graded dose‐response relationships. Evaluation of the joint associations of SES and CRF with HF risk showed that increased CRF levels appeared to attenuate the increased risk of HF associated with low SES. However, formal tests showed no significant evidence of interactive effects of SES and CRF on the long‐term risk of HF, suggesting the effect of each exposure on HF risk may be independent of the other. Given the low sample size and event rates in the exposure categories, studies with larger samples are needed to confirm or refute potential interactive effects of SES and CRF on HF risk. Finally, our findings suggest that the NNT for high aerobic fitness levels and high SES to prevent a HF event over long‐term follow‐up ranged from 6 to 35 in approximately healthy middle‐aged to older men.

The interaction between SES and HF has been reported to be complex and the precise mechanisms accounting for the association between low SES and increased HF risk remain elusive.^4^ Socioeconomic differences in potential aetiological risk factors such as alcohol consumption, hypertension and systemic inflammation, have been reported to contribute to the risk. Social deprivation is also associated with lower rates of treatment, dose and adherence to therapy for, and delayed presentation of hypertension, diabetes and CHD,^4^ which consequently lead to HF. Psychosocial factors such as stress and depression, which are strongly associated with cardiovascular outcomes, also disproportionately affect individuals of low SES.^3^ Though CRF is determined by many non‐modifiable factors such as age, sex and heritability, it remains a modifiable risk factor. The most established methods of increasing CRF are via exercise training and increased PA.^9^ Greater PA and exercise reduce HF risk through various mechanisms including (i) reducing the prevalence of standard and novel cardiovascular risk factors such as hypertension, obesity, blood glucose and coronary artery disease; (ii) preventing adverse changes in cardiac structure and function; (iii) promoting physiologic remodelling and (iv) improving cardiac, neurohormonal, skeletal muscle, pulmonary, renal and vascular performance.^5^

These findings may have important clinical implications. They add to the overwhelming evidence on the benefits of high CRF levels (via regular aerobic PA) on chronic diseases and their potential ability to attenuate the adverse effects of traditional risk factors. Despite guideline recommendations and population‐wide strategies to promote PA levels, most populations do not achieve general PA recommendations. Populations at high cardiovascular risk including the socioeconomically deprived need more education on the substantial benefits of PA. Furthermore, there should be widened access to PA resources that are both feasible and attractive for these populations.

This is the first evaluation of the separate and joint associations of SES and CRF with HF risk. We also assessed the nature of the dose‐response relationships of the exposures with HF risk. Other strengths of this analysis included the use of a prospective cohort design with exclusion of men with pre‐existing HF, the long‐term follow‐up duration of the cohort and the use of a gold standard measure of CRF. Limitations deserving consideration included the relatively low sample size due to the categorization of exposures, use of self‐administered questionnaires in assessing SES, findings may only be generalizable to middle‐aged and older northern European men and potential for biases such as residual confounding and regression dilution bias.

## 5 CONCLUSION

In a general male Finnish population, both SES and CRF were each independently associated with HF risk, potentially consistent with graded dose‐response relationships. High levels of CRF may attenuate the increased risk of HF due to low SES, but further study is needed to confirm if there are true interactive effects of SES and CRF on the long‐term risk of HF.

## CONFLICT OF INTEREST

No potential conflict of interest was reported by the authors.

## AUTHOR CONTRIBUTION

S.K.K.: Study design, data analysis and interpretation, drafting manuscript, and revising manuscript content and approving final version of manuscript; S.Y.J.: Study design and revising manuscript content and approving final version of manuscript; T.H.M: Study design and revising manuscript content and approving final version of manuscript; J.A.L.: Study design and conduct, responsibility for the patients and data collection, and revising manuscript content and approving final version of manuscript.

## Supporting information

App S1
